# Ureterocolic Fistula Involving a Native Kidney and the Sigmoid Colon in a Renal Transplant Recipient

**DOI:** 10.7759/cureus.52562

**Published:** 2024-01-19

**Authors:** Fae B Kayarian, Sarah Dynia, Anne P Timmermann, Jennifer Lee, Oyedolamu Olaitan

**Affiliations:** 1 College of Medicine, Rush Medical College, Rush University Medical Center, Chicago, USA; 2 Section of Abdominal Transplantation, Department of Surgery, Rush University Medical Center, Chicago, USA

**Keywords:** renal transplant, sigmoid colon, native ureter, native kidney, ureterocolic fistula

## Abstract

A ureterocolic fistula in a renal transplant patient is a rare complication. Even rarer is a ureterocolic fistula involving a native kidney in a patient with a functional, ipsilateral transplanted kidney, with no prior cases published in the English literature. In the current case report, we describe a patient with a previously successful right renal transplant, who presented three years later with a ureterocolic fistula involving the right native kidney and the sigmoid colon.

## Introduction

A ureterocolic fistula (UCF) is a relatively rare complication in which abnormal communication forms between a portion of the ureter and the colon. While a UCF is typically caused by obstructing ureteral calculi, other etiologies include colonic diverticular disease, Crohn’s disease, radiation therapy, malignancy, tuberculosis, or iatrogenic trauma [1, 2]. The left ureter is involved in 75% of cases, given its proximity to the sigmoid colon [3]. The development of UCF in a patient with a history of renal transplant is even rarer, with only one previous case documenting a 57-year-old female with three previously failed renal transplants who developed a fistula between a nonfunctioning renal transplant ureter and the sigmoid colon in the setting of diverticulitis [4]. We present a case of UCF involving the right native, nonfunctional ureter and the sigmoid colon in a patient with a history of successful right renal transplant. To our knowledge, this is the first published case in the English literature that describes a UCF involving a native kidney and colon in a patient with a functioning, ipsilateral renal transplant.

## Case presentation

The patient is a 72-year-old male with a significant past medical history of coronary artery disease, atrial fibrillation, right cecal villous adenoma status post right hemicolectomy, and end-stage renal disease (ESRD) status post right cadaveric renal transplant in October 2020. Following the renal transplant, the patient had multiple admissions in December 2020 for persistent pyelonephritis and ureteral stricture of the transplanted kidney, requiring percutaneous nephroureteral catheter placement. In May 2021, the patient was admitted for a ureteral stent exchange, where he was found to have an impassable distal transplant ureteral stricture and transplant pyelonephritis secondary to enterococcus and pseudomonas. He received IV antibiotics and underwent recanalization of the transplant ureter with repeat placement of percutaneous nephroureteral catheter. In June 2021, the patient was admitted for urosepsis secondary to recurrent transplant pyelonephritis. In July 2021, two attempts were made for successful percutaneous nephroureteral catheter conversion. Ultimately, a ureteropyelostomy was performed in December 2021; the right native ureter was ligated without a nephrectomy and the distal end was anastomosed to the transplant renal pelvis.

He was transferred from an outside hospital in May 2023 for a presumed diagnosis of emphysematous pyelonephritis of the right native kidney. A CT of the abdomen and pelvis (CTAP) demonstrated right native kidney hydroureteronephrosis with air and soft tissue mass in the mid-abdominal/pelvic region with sigmoid colon compression. A biopsy of the soft tissue mass revealed fibro-adenomatous tissue with culture-growing Pseudomonas aeruginosa and Streptococcus constellatus, treated with a course of antibiotics. 

In July 2023, the patient presented to an outside hospital with a four-day history of abdominal pain and shortness of breath and was admitted to the ICU for multiple metabolic derangements and acute respiratory distress requiring intubation. A CTAP demonstrated severe hydronephrosis of the right native kidney with ureteral dilation, wall thickening, and a moderate amount of gas in the right renal pelvis and ureter. Fluid and air collection adjacent to the sigmoid colon was detected, suggestive of contained perforation near the renal allograft. A nephrostomy tube placed in the right native kidney drained fecal matter, further supporting the diagnosis of a right ureterocolic fistula.

**Figure 1:**
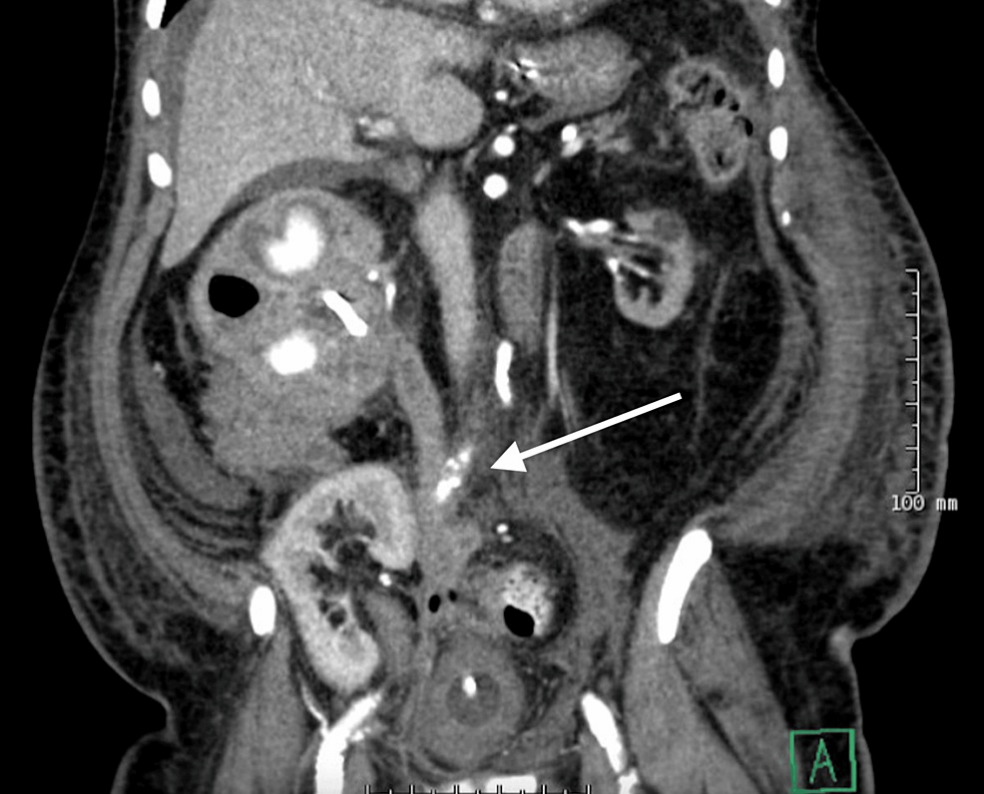
CT of the abdomen and pelvis with findings concerning for a focal sigmoid colon perforation with a right ureterocolic fistula involving the right native ureter

The patient was emergently transferred to our surgical ICU. He remained intubated, required two vasopressors, was continued on antibiotics for septic shock, and was started on continuous veno-venous hemofiltration for the treatment of acute kidney failure. Repeat CTAP demonstrated findings concerning for a focal sigmoid colon perforation with a UCF involving the native ureter (Figure 1). He underwent exploratory laparotomy with enterolysis and diverting loop ileostomy creation, with significant clinical improvement. His post-operative course was complicated by stress cardiomyopathy and delirium. He was discharged home on postoperative day 25 with plans for definitive management with a right native nephrectomy and possible bowel resection, as he continues to progress.

## Discussion

Formation of fistulas between the colon and the genitourinary system is rare, most often represented by colovesical (colon and bladder) and colovaginal (colon and vagina) fistulas [5]. A UCF is extremely uncommon and typically forms in the setting of diverticular disease, malignancy, obstructing urolithiasis, Crohn’s disease, iatrogenic trauma, or malignancy [2]. Of those cases, none have been documented to involve the native kidney ureter in a renal transplant recipient with hydronephrosis from ligated native ureter post ureteropyelostomy. A UCF is typically detected using urinary symptoms, most often presenting with symptoms including pneumaturia, fecaluria, abdominal pain, and symptoms that mimic a urinary tract infection [6]. The diagnosis is guided by symptoms and made using retrograde pyelogram, excretory urography which shows contrast material as it enters the bowel, or barium enema which shows contrast media entering the affected ureter [2]. However, if a patient presents with a UCF in a native, nonfunctional kidney with a history of renal transplant, the patient may not present with classic genitourinary symptoms and may not have a work-up that includes the typical imaging modalities used to detect a UCF. Therefore, the diagnosis may be delayed and there may be a greater chance of decompensation, colonic perforation, or urosepsis. The management of a classically presenting UCF is focused on maintaining the functional status of the kidney, which usually involves sparing any intervention to the kidney and resecting the affected bowel [2,3]. Rarely, cases will be so severe as to warrant definitive management, such as a diversion or nephrectomy [2]. 

The patient presented in this report demonstrated the importance of monitoring for a potential UCF in a nonfunctional native kidney in a post-renal transplant patient. This patient had an extensive history of recurrent pyelonephritis of the native kidney after the ureteropyelostomy, which we argue should have triggered a series of surveillance tests for UCF detection. Given that a UCF involving a native kidney in a post-renal transplant patient has virtually no literature describing it, there is no guidance or protocol for patients such as those who have recurrent infections and may be at risk of developing a UCF in their native kidney; however, prior history of colon resection, and ureteral ligation with hydronephrosis, as in this patient, should raise a high index of suspicion. While this patient’s medical and surgical management was eventually successful in treating his urosepsis, early detection of his UCF and the knowledge of symptoms for UCF involving his native kidney may have prevented him from decompensating and requiring ICU admission. Further discussion of UCFs in patients post renal transplant is required in order to advance care for patients with this condition. Given that these patients may present with atypical symptoms that do not prompt traditional imaging modalities that aid in diagnosis, expanding our knowledge about risk factors and etiology of this condition in this patient population is extremely important. With medical and surgical advances in renal transplantation, the population of successful post-renal transplant patients will continue to age and grow, which will increase the probability of this atypical presentation of UCF. Advances in detection, medical treatment, and surgical intervention for these patients are therefore crucial and may require innovations in our current imaging paradigms and diagnostic protocols.

## Conclusions

A UCF is a rare phenomenon that typically occurs in patients with a history of ureteral calculi, diverticular disease of the colon, malignancy, or trauma. This report illustrates the first documented case of a UCF in a previously ligated/hydronephrotic ureter status post ureteropyelostomy in a renal transplant recipient. In those with previous bowel or transplant surgery with multiple post-transplant complications, having a high index of suspicion for UCFs is important. Although an atypical presentation, the risk of severe complications associated with UCFs warrants close monitoring.
